# Effect of *Helicobacter pylori*–induced gastric cancer on gastrointestinal microbiota: a narrative review

**DOI:** 10.3389/fonc.2024.1495596

**Published:** 2025-01-10

**Authors:** Mohsen Heidary, Sousan Akrami, Tohid Madanipour, Nafiseh Hosseinzadeh Shakib, Marzie Mahdizade Ari, Masoumeh Beig, Saeed Khoshnood, Roya Ghanavati, Monireh Bazdar

**Affiliations:** ^1^ Leishmaniasis Research Center, Sabzevar University of Medical Sciences, Sabzevar, Iran; ^2^ Department of Microbiology, School of Medicine, Tehran University of Medical Sciences, Tehran, Iran; ^3^ Department of Microbiology, School of Medicine, Shahid Beheshti University of Medical Sciences, Tehran, Iran; ^4^ Department of Bacteriology and Virology, School of Medicine, Shiraz University of Medical Sciences, Shiraz, Iran; ^5^ Department of Microbiology, School of Medicine, Iran University of Medical Sciences, Tehran, Iran; ^6^ Department of Bacteriology, Pasteur Institute of Iran, Tehran, Iran; ^7^ Clinical Microbiology Research Center, Ilam University of Medical Sciences, Ilam, Iran; ^8^ School of Medicine, Behbahan Faculty of Medical Sciences, Behbahan, Iran; ^9^ School of Medicine, Razi Hospital, Ilam University of Medical Sciences, Ilam, Iran

**Keywords:** *Helicobacter pylori*, gastric cancer, bacterial interactions, gastrointestinal microbiota, mucos associated lymphoid tissue

## Abstract

*Helicobacter pylori* (*H. pylori*) infection is a typical microbial agent that interferes with the complex mechanisms of gastric homeostasis by disrupting the balance between the host gastric microbiota and mucosa-related factors, ultimately leading to inflammatory changes, dysbiosis, and gastric cancer (GC). We searched this field on the basis of PubMed, Google Scholar, Web of Science, and Scopus databases. Most studies show that *H. pylori* inhibits the colonization of other bacteria, resulting in a less variety of bacteria in the gastrointestinal (GI) tract. When comparing the patients with *H. pylori–*positive and *H. pylori*–negative GC, the composition of the gastric microbiome changes with increasing abundance of *H. pylori* (where present) in the gastritis stage, whereas, as the gastric carcinogenesis cascade progresses to GC, oral and intestinal-type pathogenic microbial strains predominate. *H. pylori* infection induces a premalignant milieu of atrophy and intestinal metaplasia, and the resulting change in gastric microbiota appears to play an important role in gastric carcinogenesis. The effect of *H. pylori*–induced GC on GI microbiota is discussed in this review.

## 1 Introduction

*Helicobacter pylori* (*H. pylori*) infection causes chronic gastritis, which can progress to severe gastroduodenal pathologies, including peptic ulcer, gastric cancer (GC), and gastric mucosa–associated lymphoid tissue (MALT) lymphoma (1). *H. pylori* is usually transmitted in childhood and persists for life if untreated. The infection affects around half of the population in the world, but prevalence varies according to location and sanitation standards (2). *H. pylori* has unique properties to colonize gastric epithelium in an acidic environment. The pathophysiology of *H. pylori* infection is dependent on complex bacterial virulence mechanisms and their interaction with the host immune system and environmental factors, resulting in distinct gastritis phenotypes that determine possible progression to different gastroduodenal pathologies (3). The causative role of *H. pylori* infection in GC development presents the opportunity for preventive screen-and-treat strategies (4). Invasive, endoscopy-based, and non-invasive methods, including breath, stool, and serological tests, are used in the diagnosis of *H. pylori* infection. Their use depends on the specific individual patient history and local availability (5). *H. pylori* treatment consists of a strong acid suppressant in various combinations with antibiotics and/or bismuth (6). The dramatic increase in resistance to key antibiotics used in *H. pylori* eradication demands antibiotic susceptibility testing, surveillance of resistance, and antibiotic stewardship (7).

GC, which refers to the occurrence of cancer in the stomach cell line, is one of the leading causes of cancer-related deaths and ranks as the fifth most common cancer globally, posing a significant global health challenge (8). GC, being multifactorial cancer, has risk factors that include a family history of GC in first-degree relatives, dietary habits, gender, age, race, *H. pylori* infection, nutritional status, and a history of invasive diseases such as lymphoma- or gastric-related procedures (9, 10). Studies have suggested the potential role of *H. pylori* and other bacterial genera in the progression of GC (11, 12). *H. pylori*, as a destructive member of the gastric microbiota, raises global health concerns due to its association with GC (13). This bacterium recruits neutrophils and lymphocytes to the gastric mucus, stimulating the production of reactive oxygen species (ROS) and inflammatory cytokines through the action of the CagA (cytotoxin-associated gene A) protein. This process gradually stimulates cell proliferation, leading to the development of GC and alterations in the composition of the gastrointestinal (GI) tract microbiota (14–18).

This review represents, to the best of our knowledge, the first comprehensive analysis of the interaction between GC and *H. pylori*, focusing on their impact on the microbiota. The current study highlights variations in microbiota alterations among individuals with GC, with or without *H. pylori* infection. Investigating the correlation between the presence of *H. pylori* and microbiota changes in patients with GC is crucial for developing more effective therapies for *H. pylori* infection in individuals with GC who are *H. pylori*–positive. Distinct differences exist between individuals with GC who harbor *H. pylori* and those who do not. Through multi-omics studies that analyze changes in microbial profiles and metabolite alterations, a diverse range of compositions in the GI tract microbiota has been observed in patients with GC depending on the presence or absence of *H. pylori*. In fact, individuals with *H. pylori*–positive GC exhibit very low variation in gastric microbiota and significant changes in GC microbiota, including *Firmicutes*, *Proteobacteria*, *Bacteroides*, *Streptococcus*, *Lactobacillus*, *Escherichia*, and *Shigella.* Eradicating *H. pylori* eventually restores the disrupted microbiota in patients with GC (19). Conversely, patients with GC without *H. pylori* are closely linked to high microbial diversity in the gastric microbiota, a significant increase in *Haemophilus* and *Streptococcus*, and an elevation in the abundance of metabolites (20–23). All these changes may be due to the inhibitory effects of *H. pylori* on the colonization of other microorganisms (24, 25). In addition, the four most dominant strains of gut microbiota in individuals with GC with *H. pylori* strains, including *Enterococcus*, *Escherichia-Shigella*, *Bacteroides*, and *Lactobacillus*, contribute to the progression of GC by increasing damage to cancerous tissue (26–28), the levels of tumor necrosis factor–alpha (TNF-α) (29), and unfavorable metabolites (30). Although the relationship between microbiota and metabolites in individuals with GC with *H. pylori* has not been thoroughly described, it is evident that the secondary metabolites produced by *H. pylori* elevate the metabolism of citric acid and carbohydrates in the gastric tissue of patients with GC (31), exacerbate inflammation in the gastric tissue by stimulating the activation of C-type lectin receptors (32), and inhibit the interferon-alpha (IFN-α) signaling pathway to evade the immune system (33). The importance of analyzing the differences in metabolic activity between GC cases with and without *H. pylori* reflects their potential to serve as candidate markers for distinguishing which patients with GC harbor *H. pylori* or not (20). In addition to alterations in microbiota and metabolic changes, studies indicate histopathological changes such as peptic ulcers and epithelial changes in the gastric mucosa of patients with GC with *H. pylori*. As mentioned regarding the metabolic changes, these histopathological changes are suitable indicators to determine the presence of *H. pylori* in GC cases. These histopathological changes were not present in the gastric tissue of patients with GC who were not colonized by *H. pylori* (34). The primary aim of this study was to review the effect of *H. pylori*–induced GC on GI microbiota.

## 2 Search strategy

We collected original and review articles in this field by searching through PubMed, Google Scholar, Web of Science, and Scopus databases for English language literature published up to 2024. The search was conducted on the basis of “Gastric or stomach cancer,” “*Helicobacter pylori*–induced gastric cancer,” “*H. pylori* eradication” AND “Gut microbiota,” or “Microbiome” as keywords. Studies that reported the role of *H. pylori*–induced GC on GI microbiota and changes in gastric microbiota following successful *H. pylori* eradication were enrolled.

## 3 Gastrointestinal microbiota in patients with gastric cancer

Microbial diversity and composition change in GC (35). Studies suggest that the diversity and composition of the gastric microbiota differ among patients at different histological stages of GC (11, 36). This issue emphasizes that the imbalance of the gastric microbiota is dynamic. The GC microbiome appears to improve with oral and intestinal bacterial taxa (37). Bacterial genera such as *Lactococcus*, *Bacillus*, *Prevotella*, *Veillonella*, *Leptotrichia* (38), *Achromobacter*, *Citrobacter*, *Rhodococcus*, *Phyllobacterium* (11), *Peptostreptococcus*, *Parvimonas*, *Slackia*, and *Dialister* (39), which commonly colonize the oral cavity, are enriched in the GC microbiota. Reports indicate diverse geographic regions where bacterial species of intestinal commensals, including *Lactobacillus* (11, 38, 39), *Streptococcaceae* (39, 40), *Staphylococcus* (38, 41), *Clostridium* (11, 38), and *Fusobacterium* (38, 39), are consistently enriched in GC. There are controversial results regarding the abundance of Streptococcus and Prevotella between GC and non-cancer patients. Studies indicate that they are both increased (38, 42) and decreased (11, 43) in the GC microbiota. The depletion of *Neisseria*, *Comamonadaceae*, *Acinetobacter* (39), *Vogesella*, and *Helicobacter* (11, 40) was identified in the GC microbiota. *Neisseria* (11) was a genus that showed a decrease in the microbiota of patients with GC from regions with low GC risk. However, *Veillonella* and *Leptotrichia* increased in relative abundance in the microbiota of patients with GC from areas with high GC risk (11, 44). Gunathilake et al. showed enrichment of *H. pylori*, *Propionibacterium acnes*, and *Prevotella copri* and a decrease in the abundance *Lactococcus lactis* in the gastric microbiota of patients with GC (45). In a study from Portugal, Ferreira et al. demonstrated that there was a significant decrease in *Helicobacter*, *Neisseria*, *Streptococcus*, and *Prevotella* and an increase in abundance *Lactobacillus*, *Citrobacter*, *Clostridium*, *Achromobacter*, and *Rhodococcus* in cancer versus non-cancer. The profile of the GC mucosal microbiota obtained from the 16S rRNA gene has shown metabolic activities and biochemistry such as carbohydrate metabolism, carbohydrate digestion, absorption (11, 38, 39), membrane transport (11, 43), and nucleotide/purine metabolism (39, 43) to be significantly increased in GC by enhancing the counts of nitrate-reducing bacteria. Consequently, the functions of nitrate reductase (NR) and nitrite reductase (NiR) are significantly enriched in the microbiota of GC subjects, aligning with Correa’s hypothesis (11, 43, 46).

## 4 Role of *H. pylori*–induced gastric cancer on gastrointestinal microbiota

During the different stages of GC, the diversity and composition of the bacterial microbiome vary significantly. The microbial complex shows a strong correlation with precancerous lesion stages such as atrophic gastritis (AG) and dysplasia. GC and precancerous lesions can be identified by harboring distinguishable bacterial taxa. Furthermore, the microbial structure changes on the basis of the site in patients with GC; for example, *Proteobacteria* are abundant in the gastric mucosa, whereas *Firmicutes* have been found abundantly in gastric juice (36). The reduction in gastric mucosal microbiota diversity, due to the widespread colonization of *H. pylori*, must be considered a determining factor in the association between gastric precancerous lesions and the gastric microbiota. A microbial model derived from *H. pylori*–positive gastric biopsies and stool samples serves as a critical predictor of precancerous lesions. This is supported by reports of lower bacterial taxa diversity in gastric biopsies from *H. pylori–*infected individuals compared to those from *H. pylori*–negative participants. Among *H. pylori*–infected individuals, there was an increased abundance (from 0.91% to 68.22%) of *Epsilonbacteraeota* (the fifth validly described class of the phylum *Proteobacteria*) and decreased levels of *Firmicutes* (27.55% to 8.18%) and *Proteobacteria* (36.53% to 13.97%). Moreover, the ratio of *Epsilonbacteraeota* remained unchanged in stool and gastric juice samples from the *H. pylori*–positive groups. Consequently, *H. pylori* is associated with differences in gastric mucosal bacterial diversity between *H. pylori*–positive and *H. pylori*–negative samples, underscoring the role of GI bacteria in the development of gastric precancerous lesions (47).

Exploring the potential mechanisms and dysbiosis of GI microbial composition GC involving *H. pylori* infection has revealed that richness indexes increase after the eradication of *H. pylori* infection, with approximately 18 microbial taxa altered in the gastric tract sample groups. Additionally, the dysbiotic microbiota in gastric mucosal biopsies correlate with advanced AG, intestinal metaplasia (IM), and dysplasia, and this dysbiosis may be reversed by eradicating *H. pylori*. Notably, a study observed the coexistence of *Helicobacter*, *Fusobacterial*, *Neisseria*, *Prevotella*, *Veillonella*, and *Rothia* in cases where *H. pylori* was absent in healthy superficial gastritis. It can be concluded that dysbiosis of microbial diversity contributes to carcinogenesis (19).

In addition to the remarkable diversification and increased interaction of GI bacterial composition following infection with resistant *H. pylori*, consideration must be given to the metabolic pathways and enrichment of infectious diseases. This aligns with the findings of the study by Liu et al. (2022), wherein energy metabolism, bacterial secretion systems, lipopolysaccharide synthesis, protein folding, and associated processing are enriched in *H. pylori*–positive groups. Furthermore, the imbalance in gastric mucosal microbiota manifests in the inhibition of beneficial bacterial growth, such as *Lactobacillus*. Patients with refractory *H. pylori* infections may be at higher risk of developing GC compared to other groups (48).

Conversely, virulent *H. pylori* strains may be crucial for gastric colonization, but not sufficient for the development of GC and ulcers. Distinct microbial communities exist not only in the lower GI tract of *H. pylori*–infected patients but also that in non-infected individuals. *Bacteroides* and *Bifidobacterium* colonize the gut tract of *H. pylori*–positive patients with lower frequency. Notably, *H. pylori*–infected patients experiencing stomachache exhibit a lower abundance of *Bifidobacterium* species, which may be directly associated with gastric ulcers and cancer (49).

The gastric microbial composition profile of patients provides insight into the dysbiotic cancer-associated microbiota. Typically, gastric carcinoma is triggered by *H. pylori* infection, which reduces acid secretion, allowing for the growth of a gastric microbiome with a different composition. This diversification exacerbates the invasion of bacteria into the gastric mucosa and leads to malignancy. By measuring alpha-diversity (α-diversity) using the Shannon index, it has been found that *H. pylori* influences patients with gastric carcinoma by decreasing the microbial population and enhancing the composition of other bacterial genera, especially intestinal commensals, in comparison to chronic cases. Overall, the gastric microbiota is dominated by five phyla: *Proteobacteria*, *Firmicutes*, *Bacteroidetes*, *Actinobacteria*, and *Fusobacteria.*

However, whereas the mentioned phyla colonize both GC and chronic gastritis, patients with gastric carcinoma exhibit an over-presentation of *Actinobacteria* and *Firmicutes*, along with a lower abundance of *Bacteroidetes* and *Fusobacteria* (11). *H. pylori* alters the overall structure and composition of the microbiota in the specific stomach microenvironment of GC. In patients with both histopathologically *H. pylori*–positive and *H. pylori*–negative statuses, there is a tendency for microbial diversity reduction (lower in *H. pylori*–positive and higher in *H. pylori*–negative cases). Dominant phyla in the gastric microbiota of *H. pylori*–positive groups in normal and peri-tumoral tissues are *Proteobacteria* and *Firmicutes* in high proportions. Bacterial composition decreases in the peritumoral and tumoral microhabitats (12). It is noteworthy that certain known oral microbiomes, such as *Parvimonas micra*, *Parvimonas stomatis*, *Fusobacterium nucleatum*, and *Gemella*, are likely associated with colorectal cancer and may contribute to GC. Reports indicate an abundance of oral microbiomes in GC. The differences in bacterial composition and interactions play a pivotal role in determining the total microbiota assemblage at each stage of gastric carcinoma. Significant changes in microbial diversity are observed in the richness of microbiota between GC, superficial gastritis, and IM, validating the presence of microbial dysbiosis in gastric carcinoma. The lack of compatibility may stem from various background factors, such as gender, age, ethnicity, and the involvement of *H. pylori*. Consequently, there are fewer interactions among gastric microbes at all stages, with notably more interactions between gastric microbes in *H. pylori*–negative samples than in *H. pylori*–positive groups. The interactions of *H. pylori* with gastric microbes are studied as co-occurring interactions with *Methylobacillus*, *Prevotella*, and *Arthrobacter*, along with co-excluding interactions with *Firmicutes* (*Ruminococcus*, *Bacillales*, and *Lactobacillus*) (39).

Gastric mucosa–associated lymphoid tissue (MALT) lymphoma is correlated with both the presence and absence of *H. pylori*. Additionally, the microbiota in patients with MALT lymphoma is observed even in the absence of *H. pylori*. The microbial composition in the gastric mucosal flora of patients with *H. pylori*–negative MALT lymphoma significantly decreases. This might suggest that the balance of bacteria in the gastric mucosa is disrupted in patients with MALT lymphoma without the presence of *H. pylori*. The genera *Burkholderia* and *Sphingomonas* are identified abundantly in patients with MALT lymphoma compared to those in control groups. Therefore, *Burkholderia* and *Sphingomonas* genera may contribute to the progression of MALT lymphoma. In contrast, the enrichment of *Prevotella* and *Veillonella* is lower (50). The weighted principal coordinate analysis demonstrates that the colonization of *H. pylori* increasingly alters the structure of five genera of microbiota (*Proteobacteria*, *Bacteroides*, *Fusobacteria*, *Actinobacteria*, and *Firmicutes*); however, it has little impact on the proportion of other members. Thus, alterations in the GC microbiota by increasing bacterial quantity and diversifying the microbial population could promote cancer-related activities (51). The composition of the gastric microbiota in patients with GC infected with *H. pylori* is shown in **Table 1**.

**Table 1:** Composition of gastric microbiota in patients with GC infected with *H. pylori*.

Author	Study group	Method	Microbiota composition	*H. pylori* positive or negative	Sample site	Reference
Sun et al. (2022)	134 HPN-GC cases:56 SG, 9 AG, 27 IM, 29 Dys, and 13 GC	16s rRNA sequencing	Gastric carcinogenesis stages IM and Dys: *Ralstonia* and *Rhodococcus* HPN-GC: *Streptococcaceae* and*Lactobacillaceae* AG to Dys: *Burkholderiaceae*	Negative	Gastric mucus Gastric Juice	(36)
Liu et al. (2021)	148 GC cases or gastric precancerous lesions	16S rRNA sequencing	AG: *Prevotella* and *Sphingomonas* IM: *Dorea*, *Caulobacter*, and *Bacteroides* IN: *Bradyrhizobium*, *Sphingomonas*, *Curvibacter*, and *Acinetobacter*	Negative Positive	Gastric biopsy Gastric Juice Stool sample	(47)
Guo et al. (2019)	1) 57 subjects (failed *H. pylori* treatment) 2) 58 successful *H. pylori* treatment	16S rRNA sequencing	AG, IM, Dys*: Helicobacter*, *Fusobacterial*, *Neisseria*, *Prevotella*, *Veillonella*, and *Rothia*	1) All positive 2) 49 negative	Gastric mucus Gastric biopsy	(19)
Ferreira et al. (2017)	81 patients with chronic gastritis (CG) 54 patients with GC	16S r RNA sequencing NGS	CG: Abundant *Helicobacter*, *Streptococcus*, *Neisseria*, and *Prevotella* GC: Citrobacter, Clostridium, Lactobacillus, Achromobacter, and Rhodoccocus	Positive	Gastric biopsy Gastric juice	(11)
Liu et al. (2018)	276 patients with GC	16S rRNA sequencing	Tumoral microhabitat: *Prevotella melaninogenica*, *Streptococcus anginosus*, *Fusobacterium*, *Selenomonas*, and *Propionibacterium acnes* Peritumoral microhabitat: *Helicobacter*, *Halomonas*, and *Shewanella*	Positive	Gastric tumor tissue	(12)
Coker et al. (2017)	205 GC, 21 SG, 23 AG, 17 IM, and 20 GC	16s rRNA sequencing	GC: Peptostreptoccus, Streptococcus *anginosus*, *Slackia*, *Gemella*, and *Fusobacterium* IM: *Pseudomonas*, *Dyella*, and *Acinetobacter* SG: *Comamonadaceae* and *Bacteriodes*	Positive or Negative	Gastric biopsy Gastric mucus	(39)
Wang et al. (2016)	212 patients with CG 103 patients with GC	16s rRNA sequencing	GC: *Lactobacillus*, *Escherichia*, *Shigella*, *Nitrospirae*, and *Burkholderia fungorum*	Positive	Gastric biopsy	(51)
Park et al. (2018)	138 patients 48 HPN-CSG 9 HPN-IM 23 HPN-GC, 14 HPP-CSG, and 12 HPP-GC	16S rRNA sequencing	HPN-CSG: *Firmicutes* and *Cyanobacteria* HPN-IM: *Rhizobiales* HPN-GC: *Xanthomonadaceae*, *Streptococcaceae*, *Moraxellaceae*, and *Pseudomonadaceae*	Positive and Negative	Gastric biopsy from gastric antrum	(52)

## 5 Impact of *H. pylori* eradication on gastrointestinal microbiota

### 5.1 Impact of H. pylori eradication on the gastric microbiome

For many years, *H. pylori* eradication has been utilized; however, the impact of this eradication on the normal stomach microbiota remains unknown. Eradicating *H. pylori* reduces the risk of GC, with this effect becoming more pronounced with age. Currently, eradication is targeted at preventing the development of GC (53). The acid-suppressive effects of proton pump inhibitors (PPIs) and the bactericidal activity of antibiotics form the basis of *H. pylori* eradication therapy. Antibiotics directly and powerfully affect all bacteria in the stomach (54). The strong acid-inhibitory action of PPIs can rapidly raise the stomach’s pH, limiting the influence of gastric acid on eradicating transient bacteria, which is not conducive to digestion and results in various fluctuations in substrate levels (55). Sung et al. reported that a 1-week combined treatment of omeprazole, amoxicillin, and clarithromycin (OAC) effectively eliminated *H. pylori*, leading to a significant increase in stomach bacterial diversity after 1 year. In the absence of *H. pylori*, there was a notable shift in bacterial co-occurrence, along with a distinct cluster of oral microorganisms. Levels of *Haemophilus*, *Neisseria*, and *Actinobacillus* were significantly reduced following OAC therapy (56). Additionally, according to Mao et al., stomach microflora diversity and relative quantities were greatly reduced following *H. pylori* infection. However, after successful eradication, the stomach microbiota might be partially restored to an *H. pylori*–negative state (57). Mao et al. also noted that, after *H. pylori* infection, there was a significant decrease in the diversity and relative quantity of stomach microflora. However, following successful eradication, the stomach microbiota may be partially restored to an *H. pylori*–negative condition (58).

*H. pylori* exhibits an inverse relationship with the diversity of stomach microbiota. Following successful eradication of *H. pylori*, the phylum and genus composition of stomach flora can be restored to levels comparable to those of *H. pylori*–negative patients, leading to an increase in the bacterial diversity index (59). *H. pylori* has an inverse relationship with the diversity of stomach microbiota. Following successful *H. pylori* eradication, the phylum and genus composition of the stomach flora can be restored to levels equivalent to *H. pylori*–negative patients, and the bacterial diversity index rises (60). Research conducted in China and Hong Kong revealed that only *H. pylori*–related taxa were significantly decreased following eradication. After eradication, *Firmicutes*, *Bacteroidetes*, *Actinobacteria*, *Cyanobacteria*, and *Fusobacteria* emerged as the most abundant taxa. These observations indicate that *H. pylori* serves as the primary disruptor of stomach commensal homoeostasis (19, 60). Notably, a significant increase in the relative abundance of *Anaerofustis* was observed 6 months after eradication, potentially due to the anti-inflammatory and antimicrobial properties of butyrate-producing bacteria. This increase may contribute to restoring the delicate balance between the human host and the perturbed microbiome (61). The long-term study underscores the potential role of stomach bacteria in the formation and maintenance of precancerous gastric lesions in the absence of *H. pylori*. These findings suggest that they could serve as therapeutic targets for the prevention of gastric carcinogenesis.

### 5.2 Impact of *H. pylori* eradication on the gut microbiome

**Figure 1:**
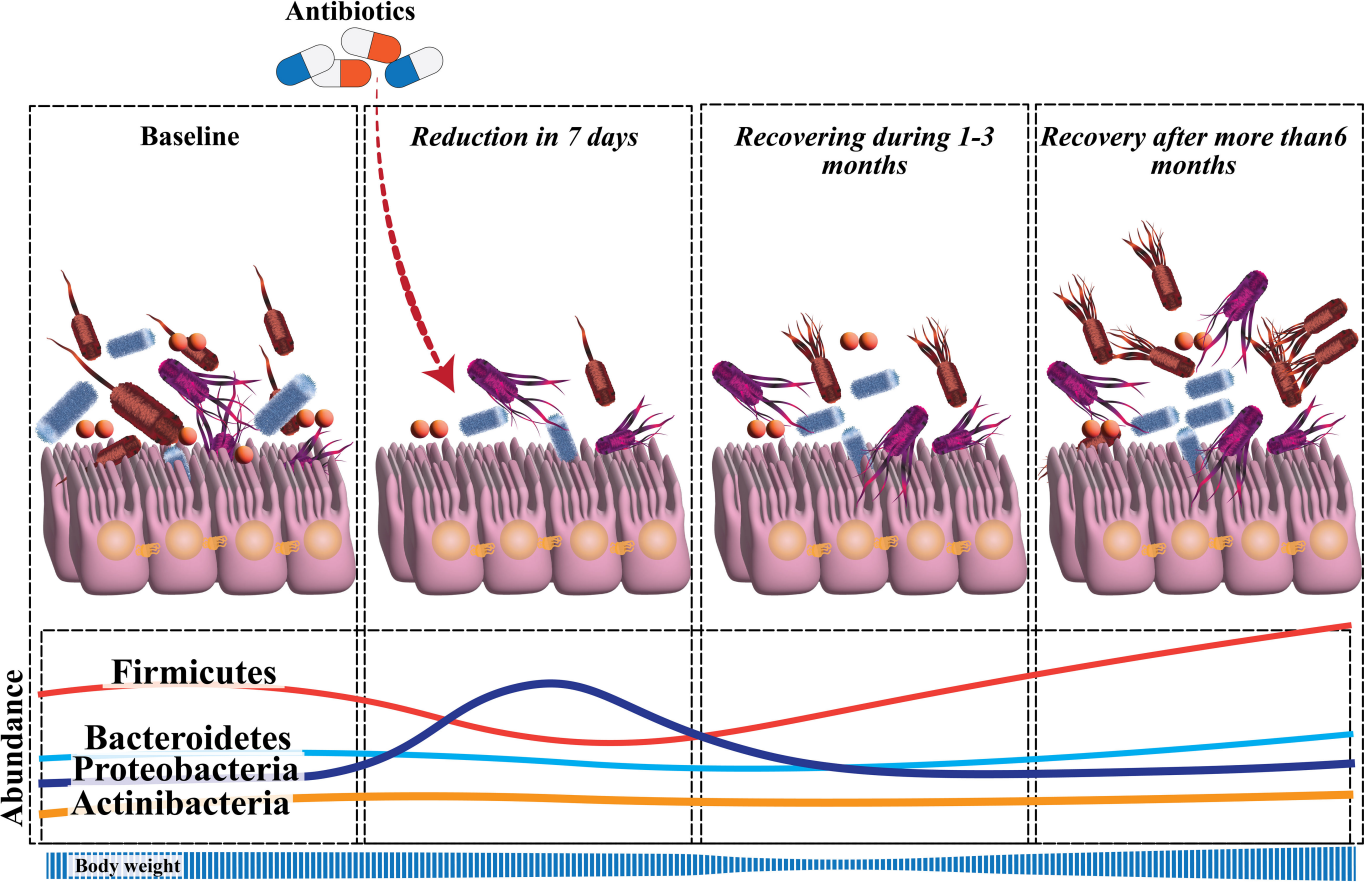
Significant perturbation of the diversity and composition of gut microbiota develops soon after *H. pylori* eradication. The microbial diversity recovers during the follow-up, but there is not yet sufficient data to confirm the changes in alpha-diversity that occur at the long-term follow-up. There is a reduction in *Actinobacteria*, relative to baseline, throughout the follow-up. *Proteobacteria* have a higher relative abundance at the short-term follow-up, which then returns to normal. Only during the long-term follow-up, a reduction in *Bacteroidetes* and a rise in *Firmicutes* were evident.

The literature on the changes in the gut microbiota caused by *H. pylori* eradication is best categorized as those that investigate immediate, short-term, and long-term impacts. The term “immediate effects” refers to those observed within 2 weeks after the treatment’s completion (62). In a study of 70 patients who underwent bismuth-based triple treatment for 14 days, it was discovered that, on day 14, α-diversity had reduced, and the Bacteroides-to-Firmicutes ratio had fallen from 0.98 to 0.3417 (63). The short-term effects of eradication treatment are those measured within 2–3 months of therapy completion (62). Short-term trials investigated triple therapy with PPI, amoxicillin, and clarithromycin, as well as bismuth-based quadruple therapy for 7 days. Three months following eradication treatment, bacterial diversity was consistently changed. Firmicutes were less common in individuals who had triple treatment, but Proteobacteria were more prevalent. Proteobacteria relative abundance rose in bismuth-treated individuals, but Bacteroidetes and Actinobacteria relative abundance decreased (63–65). Jakobsson et al. revealed that short-term antibiotic treatment for *H. pylori* eradication delivered a profound insult to the GI flora and resulted in a perturbed oral and colonic microbiome observed one week after treatment and persisting up to four years later. Short-term and long-term changes in gut microbiota after *H. pylori* eradication are reviewed in **Figure 1** (65).

The findings of a study, which indicated that the diversity of microbiota tends to decrease in the short term following eradication before returning to baseline, were consistent with the results of other investigations (63, 66, 67). Long-term studies focus on assessing the effects of eradication therapy on the gut microbiota 6 months or more after treatment. Descriptive studies have examined the long-term impacts of eradication treatment on the gut flora. By 1 year, the α-diversity and β-diversity of the microbiota, along with the relative abundance of all phyla, had returned to pre-treatment levels; however, notable alterations were observed at the genus level (61, 65, 68). More than half of the studies on the impact of *H. pylori* on the gut microbiota have entailed sub-analyses of the effects of eradication therapy on the gut microbiota (63, 66, 69). A recent comprehensive analysis of 24 studies investigating the influence of *H. pylori* eradication on the gut microbiota revealed that the majority of studies have shown a significant decrease in the α-diversity of the gut microbiota shortly after eradication, with no further changes reported beyond 6 months after *H. pylori* eradication. Additionally, *Proteobacteria* abundance increased during short-term follow-ups, whereas *Lactobacillus* abundance decreased; *Enterobacteriaceae* and *Enterococcus* abundance increased during short-term and intermediate follow-ups (70).

**Figure 2:**
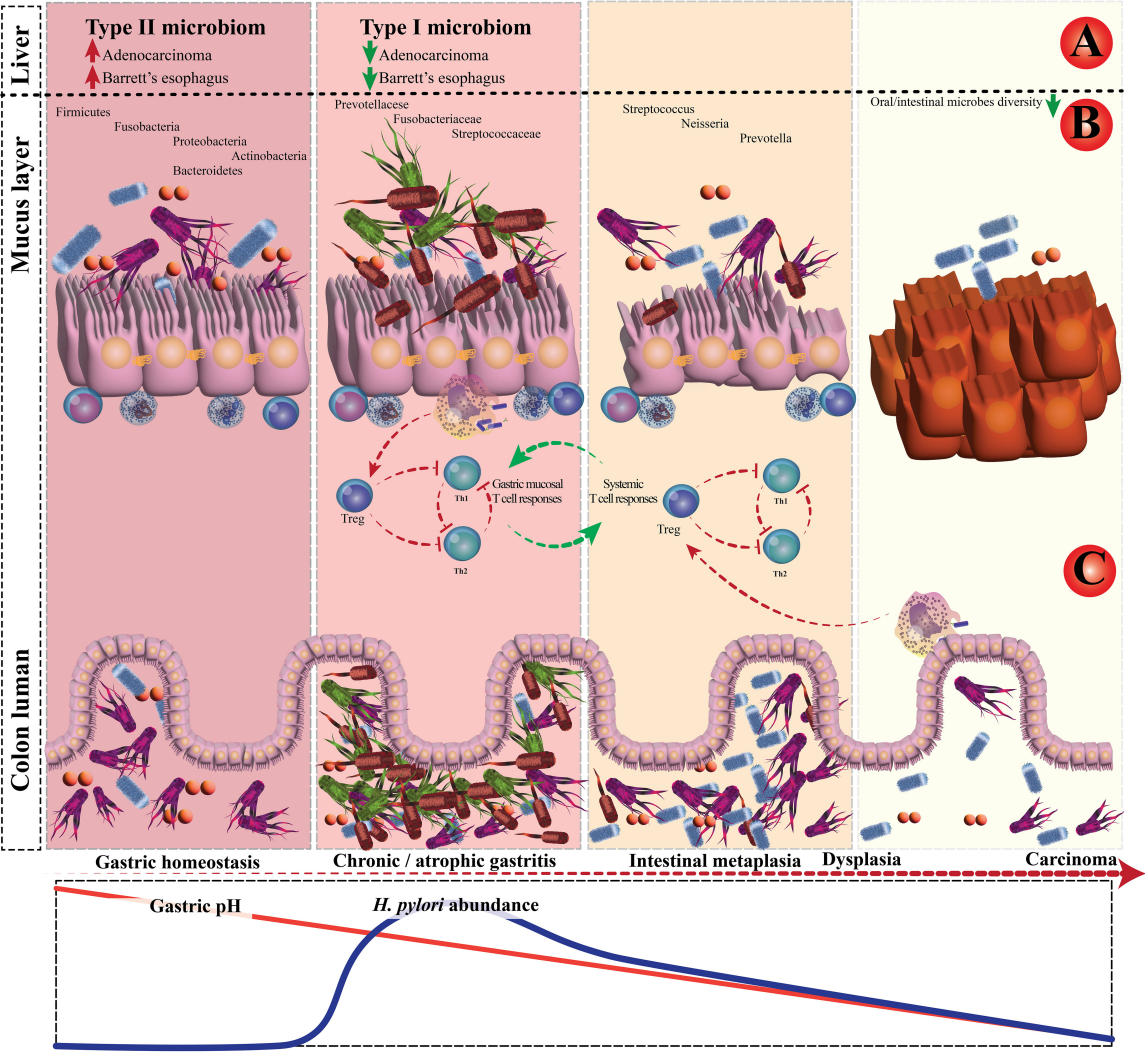
The interplay between *Helicobacter pylori* and gastrointestinal microbiota. **(A)** Case-control and epidemiology studies demonstrated that *H*. *pylori* infection is inversely associated with Barrett’s esophagus and esophageal adenocarcinoma. **(B)** Schematic plot presentation of the influence of *H*. *pylori* on gastric and colonic microbiota. **(C)** In chronic *H*. *pylori* infections, the *H*. *pylori*–experienced dendritic cells retain a semi-mature phenotype.

**Figure 3:**
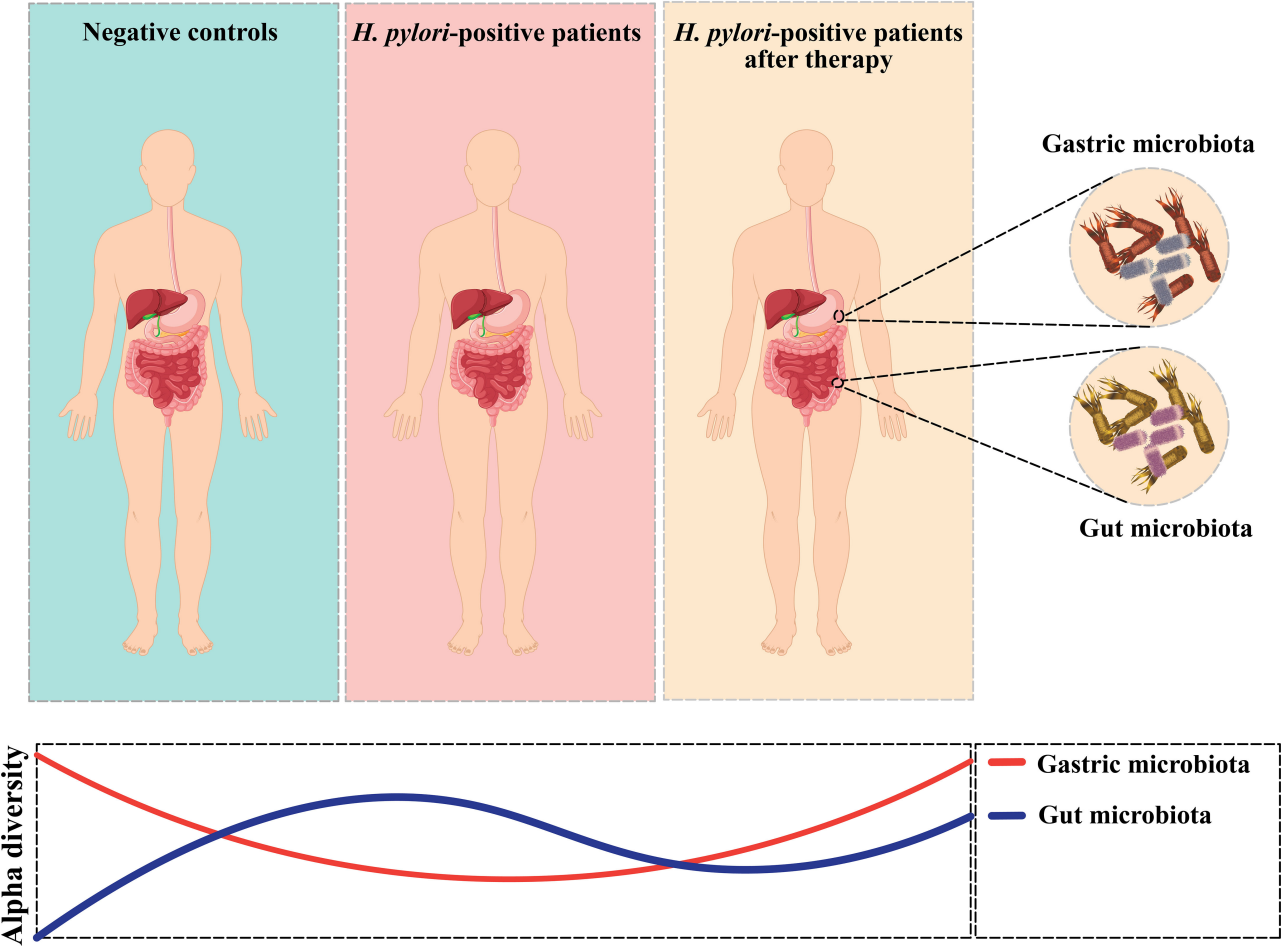
Alpha-diversity shifts in gastric microbiota and Alpha-diversity shifts in gut microbiota.

Recent research examining the long-term impacts of *H. pylori* eradication has revealed that the diversity of the gut microbiota was restored to a baseline state over the 2 years following eradication, with minimal differences in the relative abundances of microbial species at the genus level before and after eradication. However, there were slight variations in taxonomic diversity before and after eradication (71). The interaction between *H. pylori* and the GI microbiota is depicted in **Figure 2**. Additionally, according to Tao et al., the model of α-diversity shifts during *H. pylori* infection, and eradication therapy is illustrated in **Figure 3** (72). Future research should focus on investigating the microbiome over time, from pre-eradication to post-eradication and during follow-up, in relation to the development of lesions.

## 6 Effect of *H. pylori* eradication on gastric cancer prevention

Several studies demonstrate that individuals who tested positive for *H. pylori* were three to six times more likely to develop GC in comparison to uninfected controls. So, it suggested that screening for and eliminating *H. pylori* is a cost-efficient method for averting GC in individuals in their middle ages (73–75). The recognition of this bacterium as a disease-causing agent prompted certain authors to advocate for diverse programs aimed at eradicating the infection within the population, as a means of curtailing the progression of the disease (76). Many studies of randomized clinical trials (RCTs) showed that eradicating *H. pylori* leads to a decrease in GC incidence among healthy and undergone endoscopic resection of early GC (**Table 2**).

**Table 2:** Characteristics of randomized controlled trials of *H. pylori* eradication on individuals with asymptomatic infected gastric cancers and undergone endoscopic resection of early gastric cancers.

Author (years)	Country	Subjects GC/treatment vs. control	Mean age (years)/proportion of women subjects (%)	Method used to confirm presence of *H. pylori*	*H. pylori* eradication therapy regimen/ duration (days)	Eradication rate in treated/control	Follow-up period (years)	Outcome	Reference
Individuals with undergone endoscopic resection of early gastric cancers									
Fukase et al. (2008)	Japan	9/272 vs. 24/272	69 (20–79); 76.4%	Histological examination of and rapid urease testing using gastric biopsies obtained at upper gastrointestinal endoscopy	Lansoprazole at 30 mg, amoxicillin at 750 mg, and clarithromycin at 200 mg Twice a day (b.i.d.) for 7 days	75%/5%	3	Proactively eliminating *H. pylori* following the endoscopic removal of early gastric cancer is recommended to prevent the occurrence of metachronous gastric carcinoma.	(77)
Choi et al. (2018a)	Korea	18/437 vs. 36/440	60 (20–75)/67.7%	Histological examination of and rapid urease testing using gastric biopsies obtained at upper gastrointestinal endoscopy	Omeprazole at 20 mg, amoxicillin at 1 g, and clarithromycin at 500 mg b.i.d. for 7 days.	82.6%/10.5%	6	Eradicating *H. pylori* notably decreases the occurrence of MGC after endoscopic resection of gastric tumors, and it should be contemplated for *H. pylori*–positive gastric tumor patients undergoing ER.	(78)
Choi et al. (2018b)	Korea	14/194 vs27/202	59.8 (18–75)/75.3%	Histological examination of and rapid urease testing using gastric biopsies obtained at upper gastrointestinal endoscopy	Rabeprazole at 10 mg, amoxicillin at 1 g, and clarithromycin at 500 mg b.i.d. for 7 days	80.4%/5.4%	5.9	Individuals with early gastric cancer who underwent *H. pylori* treatment exhibited reduced rates of MGC and greater enhancement in the degree of gastric corpus atrophy compared to those who received a placebo.	(79)
Asymptomatic infected individuals									
Correa et al. (2000), Mera et al. (2005), and Mera et al. (2018); Piazuelo et al. (2021),	Colombia	3/437 vs. 2/415	51 (29–69)/46.1%	Histological examination of gastric biopsies obtained at upper gastrointestinal endoscopy	Bismuth subsalicylate at 262 mg, amoxicillin at 500 mg, and metronidazole at 375 mg b.i.d. for 14 days	58.0%	20	People infected with *H. pylori* and precancerous gastric lesions may gain advantages from eradication, especially individuals with atrophic gastritis lacking intestinal metaplasia.	(80–83)
Leung et al. (2004), Zhou et al. (2003), and Zhou et al. (2008)	China	2/276 vs. 7/276	52 (35–75)/48%	Histological examination and rapid urease testing	Lansoprazole at 20 mg, amoxicillin at 1000 mg, and clarithromycin at 500 mg b.i.d. for 7 days	74.5%/9.3%	10	Eliminating *H. pylori* helps protect against the advancement of premalignant gastric lesions.	(84–86)
Wong et al. (2004)	China	7/817 vs. 11/813	42.2 (35–65)/54.0	Histological examination and rapid urease testing	Omeprazole at 20 mg, co-amoxiclav at 750 mg, metronidazole at 400 mg b.i.d. for 14 days	83.7%	7.5	In a subgroup of individuals carrying *H. pylori* without precancerous lesions, eradicating *H. pylori* significantly reduced the occurrence of gastric cancer.	(87)
Saito et al. (2005)	Japan	2/379 vs. 3/313	NR (20–59)/NA	Not reported	Lansoprazole at 30 mg, amoxicillin at 1.5 g, and clarithromycin at 400 mg b.i.d. for 14 days	74.4%	≥4	*H. pylori* eradication reduced premalignant gastric lesions	(88)
You et al. (2006), Ma et al. (2012), and Li et al. (2019)	China	41/1130 vs. 78/1128	47 (35–64)/50%	Serological testing	Omeprazole at 20 mg and amoxicillin at 1 g b.i.d. for 7 days	73.2/NA	22.3	The treatment of *H. pylori* along with vitamin supplementation was also linked to a statistically significant decrease in the frequency of gastric cancer.	(89–91)
Wong et al. (2012)	China	6/510 vs. 3/514	53.0 (35–64)/46.4%	Carbon-urea breath testing	Omeprazole at 20 mg, amoxicillin at 1 g, and clarithromycin at 500 mg b.i.d. for 7 days	71.3%/NA	5	Treatment with celecoxib or eradication *of H. pylori* alone demonstrated beneficial effects on the regression of advanced gastric lesions.	(92)
Choi et al. (2020)	Korea	10/912 vs. 23/914	48.8 (40–65)/49.5%	Histological examination of and rapid urease testing using gastric biopsies obtained at upper gastrointestinal endoscopy	Lansoprazole at 30 mg, amoxicillin at 1 g, and clarithromycin at 500 mg b.i.d. for 7 days	70.1%/7.1%	9.2	For individuals with *H. pylori* infection and a family history of gastric cancer among first-degree relatives, *H. pylori* eradication treatment lowered the risk of developing gastric cancer.	(93)

## 7 Conclusion and outlook

Infection with *H. pylori*, a bacterial carcinogen, stands as the primary cause of GC, claiming hundreds of thousands of lives annually. *H. pylori* infection significantly contributes to gastric microbial dysbiosis, potentially playing a role in carcinogenesis. Successful eradication of *H. pylori* may restore the gastric microbiota to a state resembling that of uninfected individuals, thereby exhibiting beneficial effects on the gut microbiota. The current study has underscored variations in microbiota changes among individuals with GC with or without *H. pylori*. Examining the interplay between *H. pylori* infection and microbiota changes in patients with GC aids in refining therapy for *H. pylori* infection in individuals with GC and concurrent *H. pylori* presence. In the future, it is imperative to comprehensively observe changes in intestinal flora from multiple perspectives through more scientific and rational research methods. This approach will enable a thorough and clear understanding of the causes and outcomes of the relationship between GC and intestinal flora, moving beyond mere correlation analysis.
